# *Strongyloides* seroprevalence before and after an ivermectin mass drug administration in a remote Australian Aboriginal community

**DOI:** 10.1371/journal.pntd.0005607

**Published:** 2017-05-15

**Authors:** Therese M. Kearns, Bart J. Currie, Allen C. Cheng, James McCarthy, Jonathan R. Carapetis, Deborah C. Holt, Wendy Page, Jennifer Shield, Roslyn Gundjirryirr, Eddie Mulholland, Linda Ward, Ross M. Andrews

**Affiliations:** 1 Menzies School of Health Research, Charles Darwin University, Darwin, Australia; 2 School of Public Health and Preventive Medicine, Monash University and Infection Prevention and Healthcare Epidemiology Unit, Alfred Health Melbourne, Australia; 3 QIMR Berghofer Medical Research Institute, Brisbane, Australia; 4 Telethon Kids Institute, University of Western Australia and Princess Margaret Hospital for Children, Perth, Australia; 5 Miwatj Health Aboriginal Corporation, Nhulunbuy, Australia; Texas A&M University College Station, UNITED STATES

## Abstract

**Background:**

*Strongyloides* seroprevalence is hyper-endemic in many Australian Aboriginal and Torres Strait Islander communities, ranging from 35–60%. We report the impact on *Strongyloides* seroprevalence after two oral ivermectin mass drug administrations (MDAs) delivered 12 months apart in a remote Australian Aboriginal community.

**Methods:**

Utilizing a before and after study design, we measured *Strongyloides* seroprevalence through population census with sequential MDAs at baseline and month 12. Surveys at months 6 and 18 determined changes in serostatus. Serodiagnosis was undertaken by ELISA that used sonicated *Strongyloides ratti* antigen to detect anti-*Strongyloides* IgG. Non-pregnant participants weighing ≥15 kg were administered a single 200 μg/kg ivermectin dose, repeated after 10–42 days if *Strongyloides* and/or scabies was diagnosed; others followed a standard alternative algorithm. A questionnaire on clinical symptoms was administered to identify adverse events from treatment and self-reported symptoms associated with serostatus.

**Findings:**

We surveyed 1013 participants at the baseline population census and 1060 (n = 700 from baseline cohort and 360 new entrants) at month 12. *Strongyloides* seroprevalence fell from 21% (175/818) at baseline to 5% at month 6. For participants from the baseline cohort this reduction was sustained at month 12 (34/618, 6%), falling to 2% at month 18 after the second MDA. For new entrants to the cohort at month 12, seroprevalence reduced from 25% (75/297) to 7% at month 18. *Strongyloides* positive seroconversions for the baseline cohort six months after each MDA were 2.5% (4/157) at month 6 and 1% at month 18, whilst failure to serorevert remained unchanged at 18%. At 12 months, eosinophilia was identified in 59% of baseline seropositive participants and 89% of seropositive new entrants, compared with 47%baseline seronegative participants and 51% seronegative new entrants. Seropositivity was not correlated with haemoglobin or any self-reported clinical symptoms. Clinical symptoms ascertained on the day of treatment and 24–72 hrs after, did not identify any adverse events.

**Significance:**

Two community ivermectin MDAs delivered 12 months apart by trained Aboriginal researchers in collaboration with non-Indigenous researchers resulted in a sustained and significant reduction in *Strongyloides* seroprevalence over 18 months. Similar reductions were seen in the baseline cohort and new entrants.

## Author summary

We were invited by one community in East Arnhem Land to develop and deliver an ivermectin MDA to reduce the prevalence of *Strongyloides* and scabies. We demonstrated a sustained reduction in *Strongyloides* seroprevalence following the ivermectin MDA. *Strongyloides* is endemic in many Australian Aboriginal and Torres Strait Islander communities with seroprevalence ranging from 35–60%. Utilizing a before and after study design, we measured *Strongyloides* seroprevalence by ELISA through population census with sequential MDAs at baseline and month 12. *Strongyloides* seroprevalence reduced from 21% at baseline to 5% at month 6 after the first MDA. For the baseline cohort this reduction was sustained at month 12, falling to 2% at month 18 after the second MDA. For new entrants to the cohort at month 12, seroprevalence reduced from 25% to 7%.

## Introduction

*Strongyloides* is a neglected tropical disease which infects an estimated 100 million people worldwide.[1] Three species are known to parasitize humans, *Strongyloides stercoralis*, *S*. *fuelleborni* and *S*. *kellyi*.[1,2] In tropical Australian Aboriginal and Torres Strait Islander communities infection with *S*. *stercoralis* is hyper-endemic[3] with seroprevalence ranging from 35–60%.[4,5]

Strongyloidiasis can present as an acute infection with diarrhoea[6,7], bloating and intestinal distention (“pseudo-obstruction”),[8] hypokalaemia in children and wasting[3] however, many infections are asymptomatic.[3,9] Anaemia and eosinophilia have been reported in some studies[10,11] but not others.[12] Because of an auto infective cycle, asymptomatic carriage of *S*. *stercoralis* can persist for decades.[13] Complicated strongyloidiasis occurs when carriage of enteric bacteria by autoinfective larvae results in secondary sepsis or meningitis, or when the auto-infective cycle becomes uncontrolled, resulting in a large number of larvae disseminating to lungs and other organs.[1] Such dissemination is associated with high mortality[14] and most documented cases are in immunosuppressed individuals on corticosteroid therapy, or those with HIV and HTLV-1 infections.[3,15,16]

Globally there are no public health programs targeting *Strongyloides* infections however, ivermectin (the recommended treatment for *Strongyloides*) has been used in mass drug administration (MDA) programs for other parasitic infections (lymphatic filariasis and onchocerciasis) for more than 20 years.[17,18] In Australia, standard treatment guidelines target symptomatic individuals,[19] as there have been no studies providing evidence that MDA for *Strongyloides* has any impact on primary health care (PHC) presentations or in reducing population morbidity; nor is there any evidence that ivermectin MDA for chronic *Strongyloides* is effective in preventing ongoing transmission and eliminating the disease. There is however, a strong emphasis on preventing disseminated strongyloidiasis by use of pre-emptive ivermectin therapy for immunosuppressed individuals from remote Indigenous communities where *Strongyloides* is endemic.[20]

We were invited by a remote Aboriginal community to deliver an oral-ivermectin MDA targeting both *Strongyloides* and scabies. A three year regional skin health program reported no impact on scabies prevalence in children,[21] and the PHC service identified *Strongyloides* in ~25% of 300 adults screened in this community. Our aim was to determine if MDA was an effective public health measure to reduce the prevalence of both endemic infections.[22] We have previously reported the outcomes of the MDA on the prevalence of scabies;[22] here we report the outcomes against *Strongyloides* after two MDAs implemented in 2010 and 2011.

## Methods

### Ethics statement

The project was registered with the Australian New Zealand Clinical Trial Register (ACTRN– 12609000654257)[32] and received ethical approval from Human Research Ethics Committee of the Northern Territory Department of Health and Menzies School of Health Research (EC00153—project 09/34).

### Study design

Following consultation with residents from a remote island community we designed a staged roll-out of two population census and MDAs delivered 12 months apart. A survey was conducted six months after each population census to follow-up participants with equivocal or positive *Strongyloides* results and those diagnosed with scabies. The project was evaluated in a before and after study design that has been described previously.[22]

### Study location and population

The remote tropical island community is located 550km from Darwin, Australia, with an estimated population of 2000.[23]. The dry season is from March-September and the wet season from October-April with an average annual rainfall of 1400mm.[24] Temperatures range from 15–35°C and humidity from 20–95%.[24] The main language spoken is Djambarrpuyngu however, there are up to 12 other languages used in this community.[25] Most residents live in the main community however, ~200–400 resided in one of 10 associated “homelands” (small satellite settlements, five of which were accessible only by air or water). In 2010 there were 159 houses in the community at the start of the project and 165 at the second population census and MDA in 2011.

### Researchers

The project team in the community comprised of a Project Manager, Parasitologist, local Aboriginal Health Practitioners (AHPs), local Aboriginal Community Workers (ACWs), third year pharmacy students and a PhD student. The AHPs and ACWs completed a nationally accredited training program (Certificate II in Child Health Research 70131NT) delivered in community to provide them with the knowledge and skills to understand research methods relevant to the project and screen for *Strongyloides*, scabies and skin sores. Additional accredited training was provided to the ACWs so they could perform phlebotomy (units HLTPAT304B, HLTPAT306B, HLTPAT308B) and assist the AHPs and pharmacy students to administer the study medications (units HLTAP301A, CHCCS305A).

### Eligibility, recruitment and enrolment

The project commenced in the dry season and all residents were eligible to enrol. Using the Aboriginal Resource and Development community education model,[26] the ACWs and a non-Aboriginal educator visited homes and work places to provide information on *Strongyloides*, scabies, and the research project. In a subsequent visit to each house, the ACWs and/or AHPs sought and obtained written informed consent using a pictorial flipchart that incorporated a culturally appropriate process to explain the project,[27] and to also establish a household occupancy list. Parents or legal guardians provided written informed consent for children aged <18 years.

### Data and specimen collection

Portable workstations were erected at consenting participant’s homes to screen for *Strongyloides* and scabies and administer the eligible drug regimen. Implementation was over an extended period in accordance with community preference that encompassed house to house consultation, screening and treatment by the locally trained ACWs and AHPs in conjunction with pharmacy students and the project manager.

At participant’s homes, venous blood for *Strongyloides* serology was collected in a 5ml SST vacutainer, stored in insulated containers and kept cool with an ice brick. Specimens were taken to the provisional testing facility twice a day, centrifuged for 10 minutes at 3000 rpm and refrigerated overnight at 2–8°C. The SST tubes were then transported by air (2hrs) to Darwin and sorted at a commercial laboratory (Western Diagnostic Pathology) before being sent by air (4hrs) the following day to the regional reference laboratory (PathWest Laboratory Medicine) in Perth. At PathWest the samples were batched and tested weekly with the quantitative Australian *Strongyloides* ELISA in house test that used sonicated *S*. *ratti* antigen to detect anti-*Strongyloides* IgG (sensitivity 93% and specificity 95%).[28,29] The results were reported as an optical density (OD) and interpreted as seronegative (0-<0.25), equivocal (0.25–0.45) or seropositive (>0.45). Specimens were stored at PathWest for 12 months for parallel testing of subsequent specimens.

*Strongyloides* was also identified by microscopy from fresh faecal specimens within four hours of collection for those not consenting to a venous blood (mostly children). Approximately 0.005–0.01 ml of faeces was put onto a slide with normal saline under two 22mm^2^ coverslips side by side and the wet preparation examined to identify parasites. Approximately 0.2g of faeces was also inoculated onto a Mueller Hinton agar plate for culture and transported by air to the Menzies School of Health Research laboratory the following day or the next working day if collected over the weekend. Specimens were maintained between 20–27°C during transport. The agar plates once inoculated were held at room temperature for 5 days and examined on days 2, 3, 4 and 5 post-collection. On day five, the agar plate was washed with 4% formaldehyde, and examined for parasite larvae. Faecal results were reported separately (n = 80) and not included in the analysis of seroprevalence.

### Anaemia and eosinophilia

From the 6 month survey onwards, venous blood was extracted into a 4ml EDTA tube to measure haemoglobin (Hb) and eosinophil counts. After extraction the EDTA tube was inverted several times, stored in insulated containers and kept below 8°C with an ice brick. Twice a day the insulated containers were taken to the provisional testing facility where the EDTA tubes were refrigerated overnight at 2–8°C before being transported to Darwin.

Anaemia and eosinophilia were defined using the WHO haemoglobin criteria.[30] and The Royal College of Pathologists of Australasia (RCPA) reference intervals for leucocyte differential counts.[31] (Table 1).

**Table 1:** Haemoglobin (Hb)[30] and eosinophil thresholds. [31].

Age or gender group	Hb threshold (g/l)	Age	Eosinophils cell count x 10^9^/L
Children (0.5–4.9 yrs)	110	Neonate	<2.0
Children (5–11.9 yrs)	115	Children (1–3.9 yrs)	0.1–0.5
Children (12–14.9 yrs)	120	Children (4–7.9 yrs)	0.1–1.4
Non-pregnant women (≥15 yrs)	120	Children (8–12.9 yrs)	0.04–0.75
Pregnant women	110	Adult (≥13 yrs)	0.04–0.4
Men (≥15 yrs)	130		

### Ivermectin MDA and treatment of *Strongyloides*

An allocated drug regimen for *Strongyloides* was delivered based on weight and pregnancy status (Table 2). Participants were excluded from the ivermectin MDA and treatment of *Strongyloides* if they weighed <15 kg, were pregnant or females aged 12–45 years who declined a urine hCG test, had an allergy to any components of the allocated drug regimen or had received the eligible study medication in the previous seven days. Children ineligible for ivermectin received albendazole 200 mg if weight was 6-<10 kg or 400mg if weight was 10-<15 kg.

**Table 2:** Drug regimen for MDAs and treatment of *Strongyloides*.

Group	Regimen delivered at MDAs Day 1–3	Treatment at day 10–42 for those diagnosed with *Strongyloides*
**Not-pregnant**		
**<6 kg**	No MDA for *Strongyloides*	Discuss with chief investigators
**6 kg <15 kg**	200 mg (6–10 kg) or 400 mg (10-<15 kg) oral albendazole daily for 3 consecutive days	Oral albendazole 200 mg (6–10 kg) or 400 mg (10-<15 kg) daily for 3 consecutive days
**≥15 kg**	Oral ivermectin 200 μg/kg stat	Oral ivermectin 200 μg/kg
**Pregnant**	No MDA for *Strongyloides*	Treat after delivery

All non-pregnant participants who weighed ≥15 kg were administered a single dose of ivermectin 200 μg/kg at baseline and at month 12. Oral drug administration was directly observed by the researchers and given with 200mL of full-cream flavoured milk to enhance the absorption of ivermectin and albendazole. Pregnancy testing and medication administration was undertaken in portable work stations ensuring individual privacy. Treatment was repeated 10–42 days after the MDA if *Strongyloides* was diagnosed. At the month 6 and 18 surveys there was no MDA, only those diagnosed with *Strongyloides* and/or scabies and household contacts of scabies cases were treated. Participants with equivocal *Strongyloides* serology followed a treatment algorithm based on previous results (Table 3). Participants were asked a series of questions before receiving the MDA and being screened for *Strongyloides* to identify any associated symptoms, and 24–72 hrs post MDA to ascertain if any participants had experienced adverse reactions.

**Table 3:** Drug regimen for treatment of equivocal *Strongyloides* results.

	Baseline	Month 6	Month 12	Month 18
**Seen for the first time**	2 Rx*	2 Rx	2 Rx	2 Rx
**Previously negative**		1 Rx	2 Rx	2 Rx
**Previously equivocal**		No Rx if per protocol Rx at MDA#1	2 Rx if per protocol Rx not given at MDA#1 & month 6.	2 Rx if per protocol Rx not given at MDA#2.
			1 Rx if per protocol Rx given at MDA#1 & month 6.	1 Rx if per protocol Rx given at MDA#2

Rx* = 1 dose of ivermectin 200 μg/kg or 200-400mg albendazole daily for three days. 2 Rx = two doses of ivermectin or albendazole administered 10–42 days apart.

### Month 6 and 18 surveys

Two surveys were conducted during the wet season that were six months after each population census and MDA (month 6 and 18) to: a) follow-up participants with an equivocal or positive *Strongyloides* result and/or were positive for scabies in the population census six months prior, b) screen a computer-generated random sample of participants who were negative for both *Strongyloides* and scabies in the population census six months prior and c) follow-up household contacts of participants diagnosed with scabies at month 6 or 18. The staged roll-out ensured subsequent visits to households were scheduled to comply with the planned 6–12 month follow-up timeline outlined in the study protocol.[32] We estimated that a random sample of 160 participants without evidence of either disease would have a 90% power to detect an increase in the proportion of *Strongyloides* from 0 to 8%.

### Data analysis

Data were analysed using Stata 13 (StataCorp LP). *Strongyloides* seroprevalence at baseline and month 12 was calculated as a proportion of those seen who were seropositive. At month 6 and 18 surveys, seroprevalence was determined as a weighted average of (i) the failed sero-reversion rate which was calculated as the seroprevalence in participants who were seropositive at the survey and who had been seropositive at the population census six months prior, and (ii) the positive seroconversion rate which was calculated as the seropositive rate at the survey for those who were seronegative at the population census six months prior (Tables S1, S2 and S3). The OD ratio was calculated from the OD value taken six months after treatment divided by the OD value prior to treatment, as not all samples had been tested in-parallel An OD ratio >0.6 was a considered a positive seroconversion.[33]

Regression to the mean was determined through random number simulations using the same mean and standard deviation at month 0, assuming a normal distribution on the transformed scale (log of the optical density +0.01) with no decline in time irrespective of treatment, and 0.5 correlation between the optical density at month 0 and 12 as seen in the actual data.

Data entry was validated by double entering 15% of the records. The data entry error rate for variables used in the analysis was <5%.

## Results

### Enrolment for population censuses and participants seen at month 6 and 18 surveys

**Fig 1:**
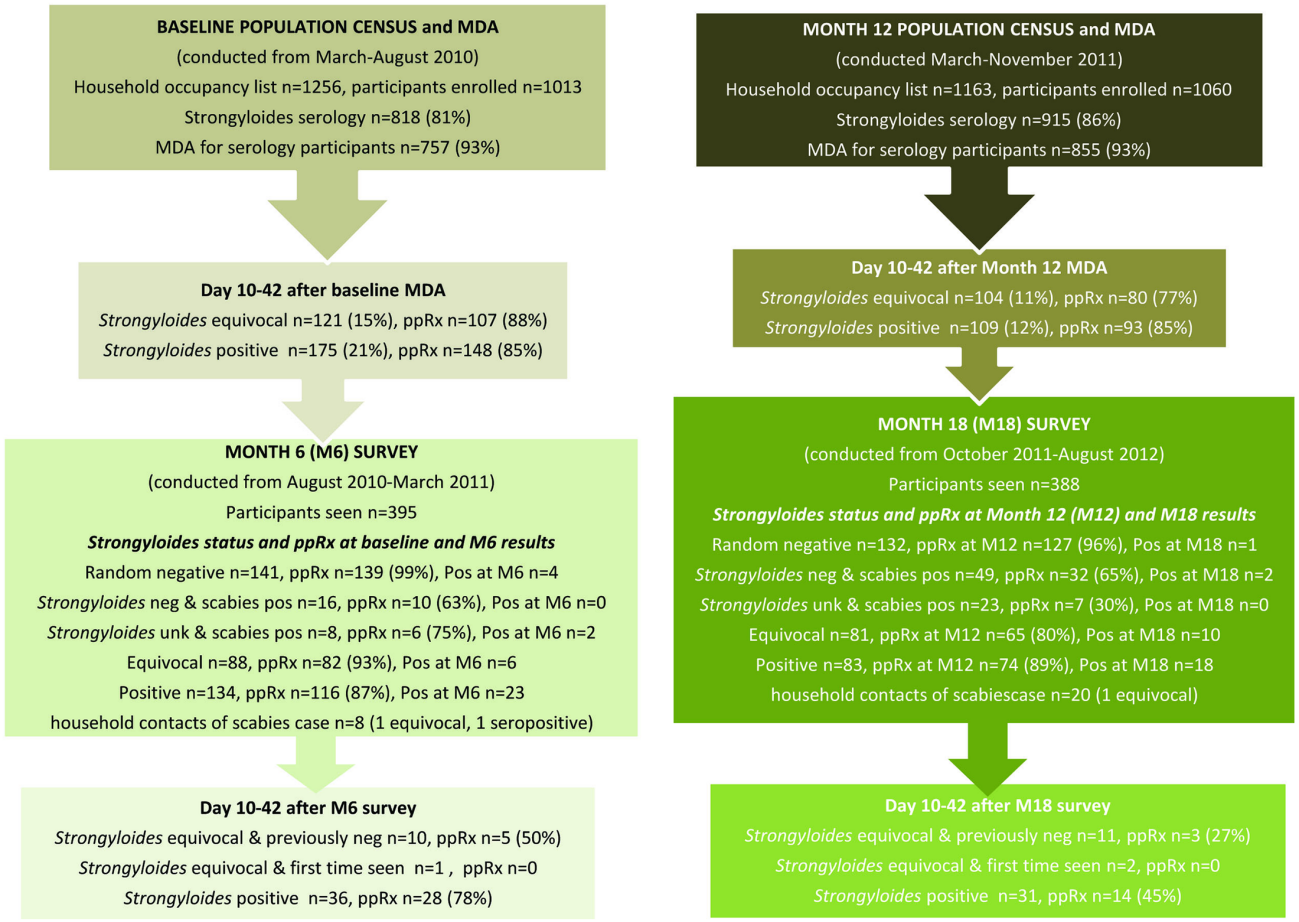
Flowchart of study design and results.

The baseline population census and MDA was conducted over six months from March-August 2010. There were 1256 residents at home at baseline of which 1013 (81%) consented to participate (Fig 1). At the month 6 survey conducted from August 2010-March 2011, 395 participants from the baseline cohort were followed up. At month 12, (March-November 2011), participation increased from 1013 to 1060 (n = 1163 residents, 91% participated) of whom 700 were participants seen at baseline and 360 were new entrants to the cohort. At the month 18 survey (October 2011-August 2012), 388 participants from month 12 were followed-up, 235 from the baseline cohort and 153 new entrants.

### Strongyloides screening

At baseline, 859 (85%) participants were screened for *Strongyloides*, 41 by faecal microscopy/culture and 818 by serology (Table 4). Of the 859 participants screened, 175 (21%) were seropositive, four (11%) were faecal microscopy/culture positive and 121 (15%) equivocal. Per protocol MDA was given to 938 (93%) participants that included 786 (92%) screened for *Strongyloides*. Ivermectin was administered to 853 (84%) participants and albendazole to 85 (8%) participants.

**Table 4:** Participant’s age, gender, treatment and haematology results by diagnostic method at baseline and month 12.

	*Strongyloides* serology			Faecal microscopy/culture		Unknown	Total
	seronegative	equivocal	seropositive	negative	positive		
*Baseline cohort*	*n = 522 (%)*	*n = 121 (%)*	*n = 175 (%)*	*n = 37 (%)*	*n = 4(%)*	*n = 154 (%)*	*n = 1013 (%)*
Median age (IQR)	28 (14–42)	29 (15–43)	21 (12–31)	3 (1–4)	3 (2–4)	5 (3–9)	21 (9–37)
0-<5 years	13 (3)	0	0	28 (76)	3 (75)	56 (36)	100 (10)
5-<15 years	122 (23)	29 (24)	62 (35)	9 (24)	1 (25)	85 (55)	308 (30)
>15 years	387 (74)	92 (76)	113 (65)	0	0	13 (8)	605 (60)
Male	230 (44)	62 (51)	99 (57)	21 (57)	2 (50)	84 (55)	498 (49)
Female	292 (56)	59 (49)	76 (43)	16 (43)	2 (50)	70 (45)	515 (51)
Scabies	19 (4)	6 (5)	8 (5)	3 (8)	0	6 (4)	42 (3)
***Per protocol treatment***							
Ivermectin	492 (94)	107 (88)	148 (85)	8 (22)	1 (25)	97 (63)	853 (84)
Albendazole	11 (2)	0	0	21 (56)	3 (75)	50 (32)	85 (8)
No treatment	19 (4)	14 (12)	27 (15)	8 (22)	0	7 (5)	75 (7)
*Month 12 –Baseline cohort*	*n = 521(%)*	*n = 63 (%)*	*n = 34 (%)*	*n = 17 (%)*	*n = 1(%)*	*n = 64 (%)*	*n = 700 (%)*
Median age (IQR)	25 (13–39)	27 (11–42)	15 (8–32)	5 (4–6)	4 (4–4)	4 (2–8)	22 (9–36)
0-<5 years	7 (1)	1 (2)	0	8 (47)	1 (100)	31 (48)	48 (7)
5-<15 years	148 (28)	20 (32)	17 (50)	9 (53)	0	25 (39)	219 (31)
>15 years	366 (70)	42 (67)	17 (50)	0	0	8 (13)	433 (62)
Male	252 (48)	30 (48)	19 (56)	10 (59)	0	37 (58)	348 (50)
Female	269 (51)	33 (52)	15 (44)	7 (41)	1 (100)	27 (42)	352 (50)
Scabies	43 (8)	7 (11)	2 (6)	1 (6)	0	10 (16)	63 (9)
***Per protocol treatment***							
Ivermectin	502 (96)	51 (81)	27 (79)	9 (53)	0	33 (52)	622 (89)
Albendazole	6 (1)	0	0	7 (41)	1 (100)	25 (39)	39 (6)
No treatment	13 (3)	12 (19)	7 (21)	1 (6)	0	6 (9)	39 (6)
***Haematology***							
Anaemia	93/494 (19%)	14/61 (23%)	7/31 (23%)	0	0	0	114/588 (19%)
Eosinophilia	198/425 (47%)	34/56 (61%)	16/27 (59%)	0	0	1/2 (50%)	249/510 (49%)
*Month 12 –New entrants*	*n = 181 (%)*	*n = 41 (%)*	*n = 75 (%)*	*n = 21 (%)*	*n = 0*	*n = 42 (%)*	*n = 360 (%)*
Median age (IQR)	24 (14–36)	25 (14–36)	16 (11–31)	2 (1–4)	-	4 (2–7)	18 (9–32)
0-<5 years	2 (1)	0	0	17 (81)	0	24 (57)	43 (12)
5-<15 years	44 (24)	11 (27)	32 (43)	3 (14)	0	14 (33)	104 (29)
>15 years	135 (75)	30 (73)	43 (57)	1 (5)	0	4 (10)	213 (59)
Male	97 (54)	22 (54)	42 (56)	10 (48)	-	18 (43)	189 (52)
Female	84 (46)	19 (46)	33 (44)	11 (52)	-	24 (57)	171 (48)
Scabies	16 (9)	3 (7)	13 (17)	5 (24)	0	13 (31)	50 (14)
***Per protocol treatment***							
Ivermectin	173 (96)	31 (76)	66 (88)	4 (19)	-	24 (57)	298 (83)
Albendazole	3 (2)	0	0	12 (57)	-	15 (36)	30 (8)
No treatment	5 (3)	10 (14)	9 (12)	5 (2)	-	3 (7)	32 (9)
***Haematology***							
Anaemia	29/170 (17%)	7/39 (18%)	12/72 (17%)	0	-	1/2 (50%)	49/283 (17%)
Eosinophilia	79/154 (51%)	21/34 (62%)	50/56 (89%)	0	-	0/1	150/245 (61%)
*Month 12—Baseline cohort not seen*	*n = 169 (%)*	*n = 33 (%)*	*n = 53 (%)*	*n = 10 (%)*	*n = 1(%)*	*n = 47(%)*	*n = 313 (%)*
Median age (IQR)	24 (12–45)	34 (20–46)	22 (13–28)	1.5 (1–3)	3	7 (4–10)	20 (10–39)
0-<5 years	3 (2)	0	0	10 (100)	1 (100)	12 (26)	26 (8)
5-<15 years	54 (32)	8 (24)	17 (32)	0	0	29 (62)	108 (35)
>15 years	112 (66)	25 (76)	36 (68)	0	0	6 (13)	179 (57)
Male	79 (47)	17 (52)	25 (47)	4 (40)	0	25 (53)	150 (48)
Female	90 (53)	16 (48)	28 (53)	6 (60)	1 (100)	22 (47)	163 (52)
Scabies	8 (5)	1 (3)	1 (2)	1 (10)	0	1 (2)	12 (4)
***Per protocol treatment***							
Ivermectin	162 (96)	26 (79)	47 (89)	0	0	31 (66)	266 (85)
Albendazole	3 (2)	0	0	7 (70)	1 (100)	8 (17)	19 (6)
No treatment	4 (2)	7 (11)	6 (11)	3 (30)	0	8 (17)	28 (9)

At month 12, *Strongyloides* screening increased by 6% to 954 (90%) participants (636 from the baseline cohort and 318 new entrants), 39 by faecal microscopy/culture and 915 by serology. There were 34 (6%) from the baseline cohort who were seropositive at month 12, one (6%) positive by faecal microscopy/culture and 63 (10%) equivocal. For new entrants at month 12, 75 (25%) were seropositive, none were faecal microscopy/culture positive and 41 (14%) had an equivocal result. Per protocol MDA was given to 989 (93%) month 12 participants that included 885 (91%) screened for *Strongyloides*. Ivermectin was administered to 920 (87%) participants and albendazole to 69 (7%) participants.

The median age of baseline seropositive participants was 21 (IQR 12–31) that was not significantly different to that of the new entrants at month 12, (16, IQR 11–31, p = 0.11). There were no significant differences in the median age or gender of new participants at month 12 and those not seen from the baseline cohort. There were 154 participants at baseline with a median age of five (IQR 3–9) and 106 at month 12 with a median age of four (IQR 2–8) for whom *Strongyloides* status was unknown as we were unable to obtain a blood or faecal sample. More males were positive than females (25% vs 18%, p = 0.015) at baseline however, this difference was not evident at month 12 for the baseline cohort (6% vs 5%) or the new cohort seen for the first time (26% vs 23%).

At month 12 there was no evidence that *Strongyloides* seropositivity had any impact on Hb when stratified by gender and age group (excluding one male aged 0–4 years) or when comparing anaemia rates between the baseline cohort and the new entrants (23% vs 17% respectively). At month 12, eosinophilia was identified in 66 (80%, 95% CI 69%, 88%) seropositive participants and 277 (48%, 95% CI 44%, 52%) who were seronegative (difference 32%; 95% CI 22%, 41%). There were significantly more new seropositive entrant participants with eosinophilia (89%) than participants seropositive from the baseline cohort (59%), difference of 30% (95% CI 10%, 50%).

**Fig 2:**
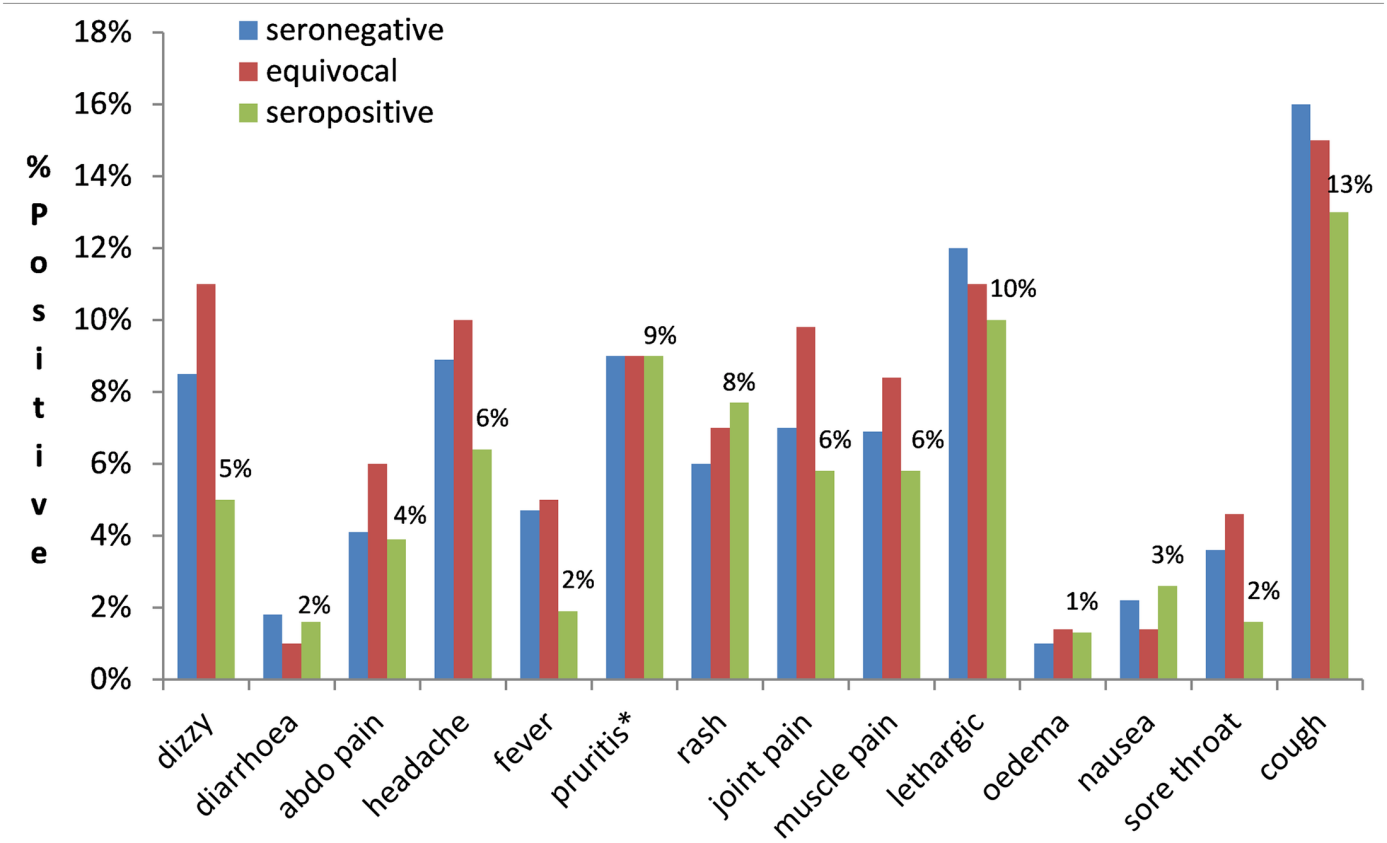
Percentage of self-reported symptoms before MDA using data from baseline, month 12 and 18 (data not collected at month 6) by *Strongyloides* serostatus. * pruritus does not include participant responses who were diagnosed with scabies and had pruritus.

From the questionnaire collected at baseline, month 12 and 18, the participant-reported symptoms on the day of MDA were not significantly associated with being seropositive, seronegative or equivocal for *Strongyloides* when stratified by age groups or analysed collectively (p>0.05 for all symptoms) (Fig 2). No adverse events after the MDAs were reported based on the self-reported questionnaire review 24–72 hrs post MDA.

### Prevalence

**Fig 3:**
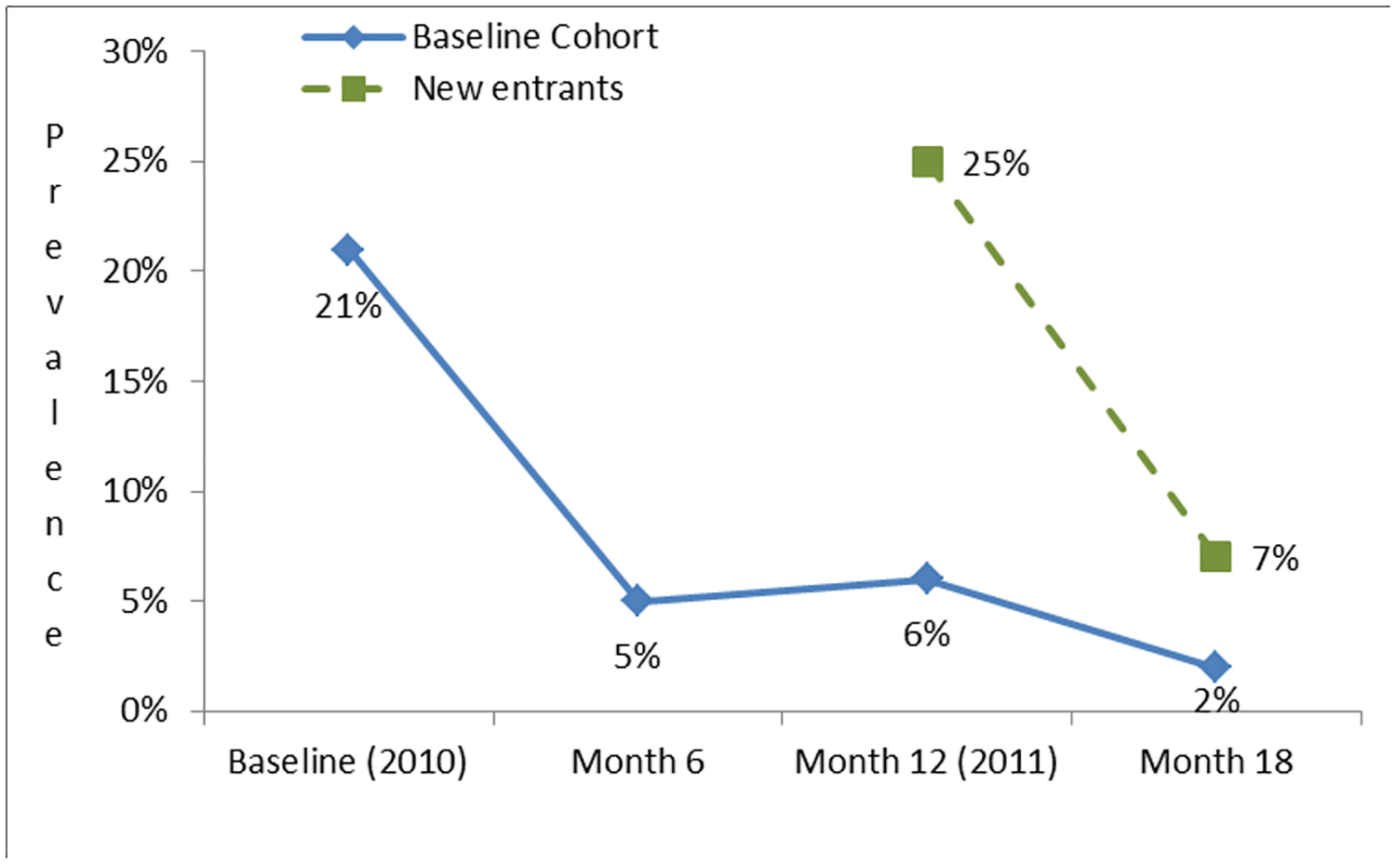
*Strongyloides* seroprevalence by month for the baseline cohort and new entrants (excludes results from those that had faecal microscopy/culture, n = 80).

*Strongyloides* seroprevalence reduced from 21% at baseline to 5% at month 6 after the first MDA (Fig 3 and Table A in S1 Data). For participants from the baseline cohort this reduction was sustained at month 12, then after the second MDA reduced further to 2% at month 18 (Table B in S2 Data). For new participants to the cohort at month 12, seroprevalence reduced from 25% to 7% at month 18 (Table C in S3 Data). The percentage of faecal specimens positive for *Strongyloides* reduced from 10% at baseline to 6% at month 12 for the baseline cohort however, the difference was not significant (p = 1).

**Fig 4:**
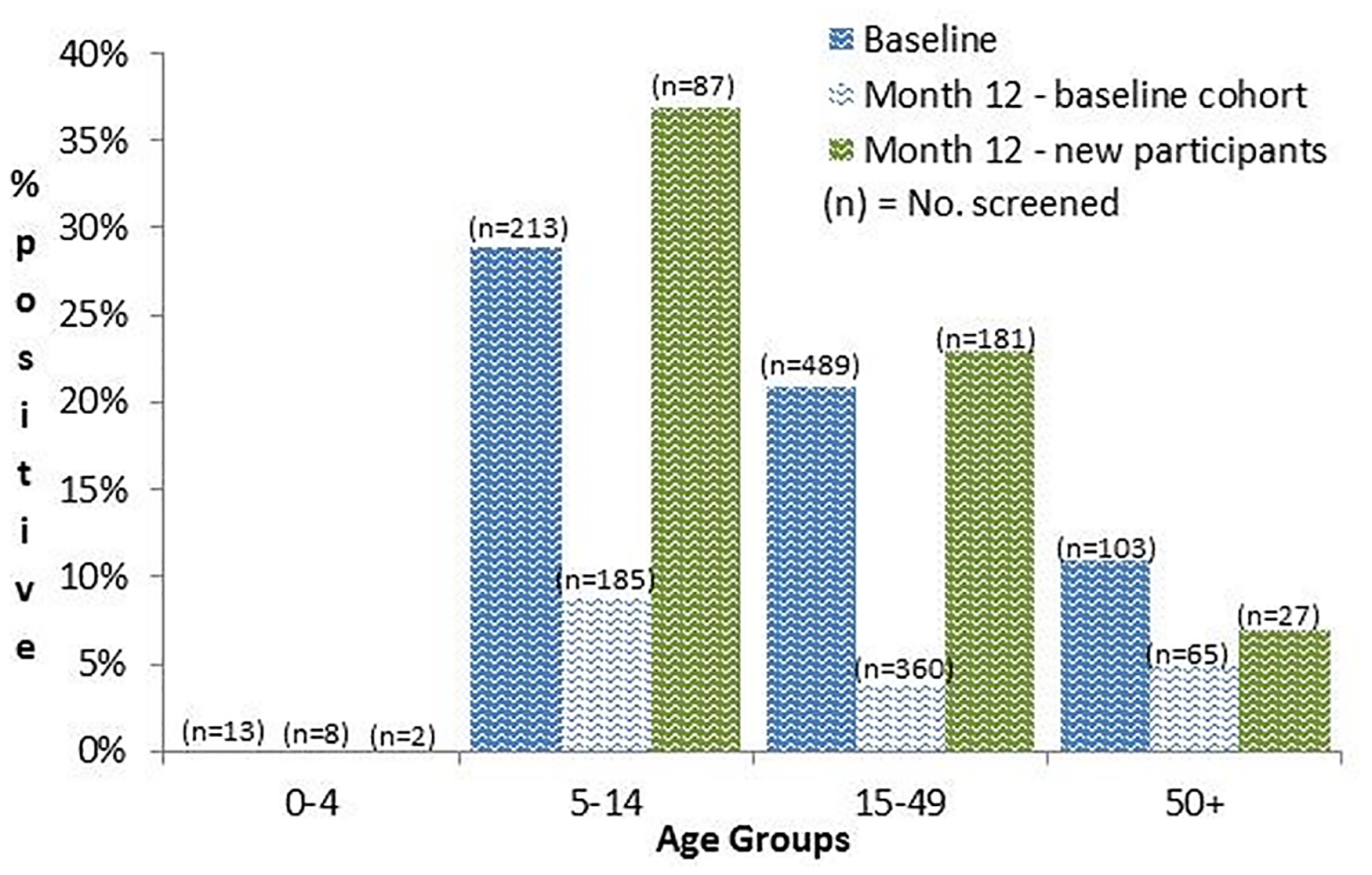
*Strongyloides* seroprevalence at baseline and month 12, by age group.

There was a high prevalence of positive *Strongyloides* serology in all age groups except those aged 0–4 years (Fig 4). Children aged 0–4 years had 23 serology tests that were all seronegative and four (7%) faecal samples that were positive by microscopy/culture. The peak serology age group at both baseline and month 12 was school children aged 5–14 years who were being seen for the first time (Fig 4). For participants seen at baseline and month 12 (n = 618), the seroprevalence reduced across all age groups.

### Participants followed-up at month 6 and 18 surveys

At month 6, we were to follow-up 460 participants, 179 that were positive for *Strongyloides* (175 seropositive and four faecal positive), 121 with equivocal results and 160 that were randomly selected participants who were negative for *Strongyloides* and scabies at baseline (Table A in S1 Data). Of the 460 participants, we followed-up 363 (79%), 133/175 (76%) that were seropositive, 1/4 (25%) that was faecal positive, 88/121 (73%) that were equivocal and 141/160 (88%) that were negative for *Strongyloides* and scabies. We also screened an additional 32 participants of which 16 had scabies but were negative for *Strongyloides* at baseline, eight had scabies but an unknown *Strongyloides* status and eight were household contacts of scabies cases diagnosed at month 6.

At month 18, there were 374 participants that required follow-up, 110 that were positive for *Strongyloides* (34 seropositive and 1 faecal positive from the baseline cohort and 75 seropositive from new entrants) 104 participants with equivocal results (63 from baseline cohort and 41 from new entrants) and 160 that were negative for *Strongyloides* and scabies at month 12 (Table 4). Of the 374 participants, 296 (79%) were seen (178 from the baseline cohort and 118 from the new entrants), 83/110 (75%) that were seropositive, 81/104 (78%) with equivocal results and 132/160 (83%) that were negative for *Strongyloides* and scabies. We also screened an additional 63 participants, 49 (30 from the baseline cohort and 10 new entrants) that were scabies positive and *Strongyloides* negative at month 12, two that had scabies but were negative for *Strongyloides* and 20 household contacts of scabies cases diagnosed at month 18.

### Positive seroconversions and failed seroreversions

At month 6, the positive seroconversion rate was 2.5% (4/157) and the failed seroreversion rate 17% (23/134) for the baseline cohort (Table A in S1 Data). Of the 134 participants seen at month 6 that were previously seropositive at baseline, 23 (17%) failed to serorevert however, all but three had an OD ratio >0.6 (S1 and S2 Figs). For the three participants that had an OD ratio <0.6 at month 6, and would be classified as failed seroreversions, the basline antibody level was only slightly above the 0.45 threshold for positive (0.47, 0.48, 0.51 respectively). Of the three participants with an OD ratio <0.6 at baseline who reverted to equivocal at month 6 (OD 0.31, 0.36, 0.42), two had received two doses of ivermectin at baseline and one had received one dose. Of the 23 participants who were seropositive at baseline and month 6, 19 (83%) had received two doses of ivermectin at baseline and the remaining four had received only one dose. The median time between ivermectin treatments for those participants failing to sero-revert was 15 days (IQR 12–21). Failure to serorevert for males was almost double (21%, n = 16) that of females (12%, n = 7) (p = 0.15). At month 18, of the 12 that had an OD ratio >0.6, five had an increase in OD and the other seven still had an OD in the positive range (>0.45).

At month 18, the positive seroconversion rate for the baseline cohort was 1% (1/127) and 4% (2/46) for the new entrants (Table B in S2 Data and Table C in S3 Data). The failed seroreversions for the baseline cohort was 32% (9/28) and 18% (9/50) for new entrants. Of the 18 that failed to serorevert, nine (50%) were new entrants of which five (56%) had an OD <0.6 (S3 and S4 Figs). Fifteen (83%) participants had received two doses of ivermectin at month 12 and the remaining three had received only one dose. The median time between ivermectin treatments for participants failing to serorevert was 23 days (IQR 17–28).

The median time interval from baseline to the survey at month 6 was 5.8 months (IQR 5–7) and 7.6 months (IQR 6–9) from the second population census at month 12 to the survey at month 18. After excluding participants that were seen at all four time points (baseline, month 6, 12 and 18) there was no difference in the failed seroreversion rate at month 6 (7/38, 18%) and month 18 (9/50, 18%).

### Serological changes

**Fig 5:**
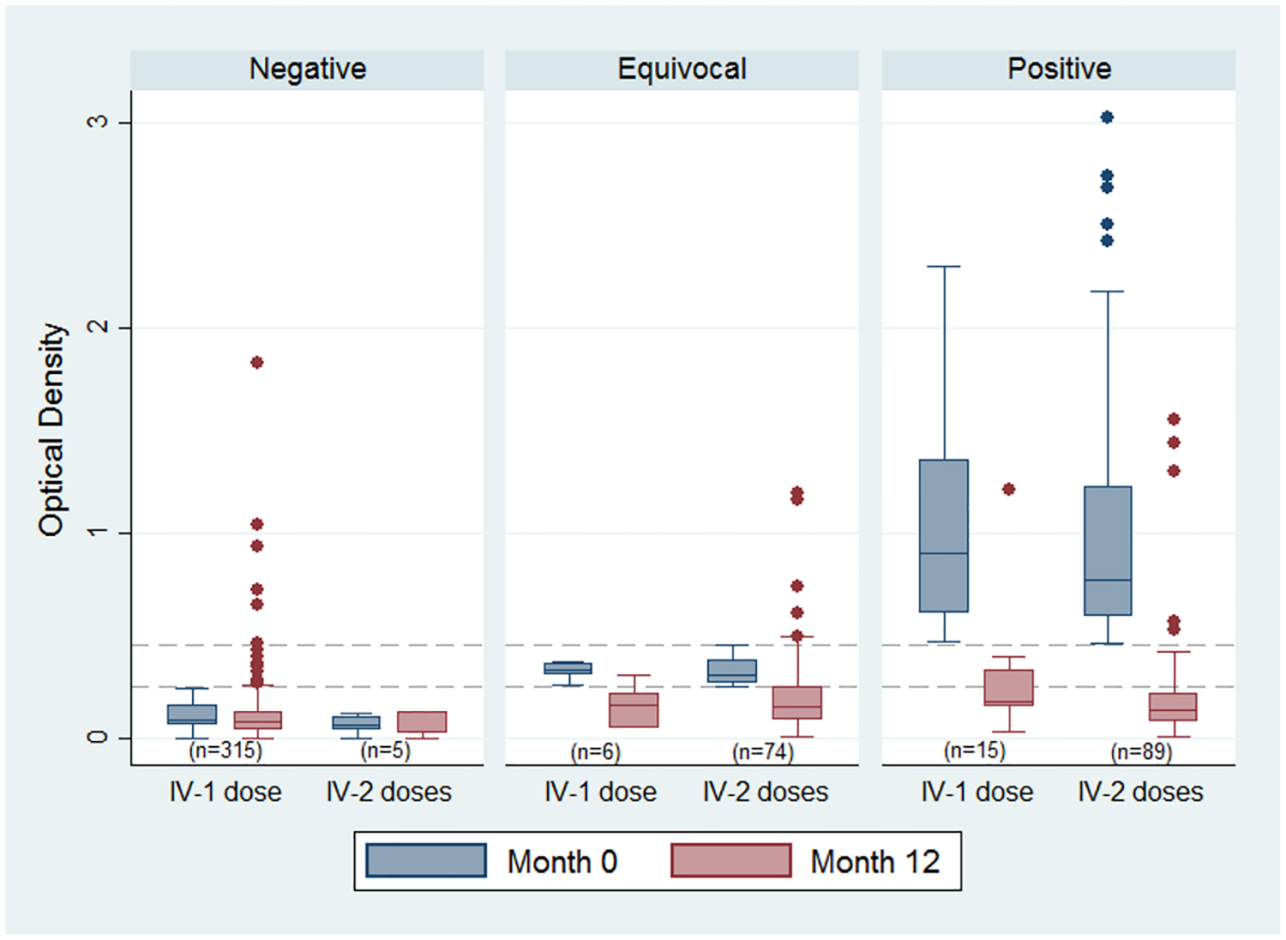
Optical density of *Strongyloides* serology for participants seen at baseline and month 12 by number of ivermectin doses and serology status (n = 504, excludes those treated at month 6).

There were 504 participants with paired serology from baseline and month 12 who did not receive ivermectin at month 6. Of the 504 participants, there was minimal difference in the median OD when comparing the number of ivermectin doses (1dose v’s 2 doses) (Fig 5). For seropositive participants receiving either one or two doses of ivermectin the median difference in the change in OD was 0.07 (p = 0.76). The median OD for those negative at baseline remained unchanged at month 12 for the majority of participants with the exception of 21 (7%) who had received only one dose and had an increase in OD above the negative cut-off of 0.25. Regression to the mean simulations showed that this phenomenon explains 50% of the reduction in OD, inferring the other 50% is attributable to MDA, under the assumption that if we had not given ivermectin there would be no change in the OD.

## Discussion

In our study, the ivermectin MDA led to a substantial reduction in *Strongyloides* seroprevalence from 21% at baseline to 5% at month 6. The lower prevalence was maintained by the baseline cohort at month 12 when there was a second MDA which further reduced prevalence to 2% at month 18. The 360 new entrants to the study at month 12 had a similar substantial reduction in seroprevalence on follow-up at month 18. In a region where temporary relocation for cultural activities is common,[34] the introduction of new entrants at month 12 highlighted the significant contribution both a repeated MDA and community ownership had in sustaining a low prevalence for the baseline cohort. The replication of baseline prevalence in the new cohort at month 12, and subsequent rapid reduction from 25% to 7% after MDA, supports the concept of incorporating a multi-faceted control program with ongoing surveillance and repeated annual MDAs at least initially, to achieve a sustainable reduction in seroprevalence.

Diagnosis of *Strongyloides* is problematic as there is no gold standard test and detection rates vary between diagnostic tools used.[15] Coprological examination tends to underestimate the prevalence of the parasite in population-based studies, and whilst serology gives a higher prevalence,[13] it often does not detect acute or hyperinfections.[29] The ELISA test was a suitable serological test for our epidemiological study where prevalence of chronic *Strongyloides* infection was high and response to treatment could be monitored through the change in optical density.[5]

At month 6, 15 (65%) participants that failed to serorevert to negative (OD <0.25) had an OD >1.0 at baseline and five (28%) at month 18. Kobayashi et al. (1994) demonstrated that OD results with extremely high antibody levels failed to serorevert to negative despite being coprologically negative and postulated that an OD ratio <0.6 was an accurate measure of failed seroreversion.[33] Twenty (87%) participants from month 6 and 12 (67%) from month 18 had an OD ratio >0.6 and would be considered positive serocoversions using Kobayashi’s ratio.

Over 80% of participants who failed to serorevert had received two doses of ivermectin, with no difference in the median time interval between doses when compared to those who did serorevert. The shorter median time interval for follow-up at month 6 of 5.1 months, may have overestimated those who failed to serorevert (17%), as serology can take six months or longer to revert to negative.[33,35] Nevertheless, there was no significant difference in the seroreversion failure rate at month 6 (18%) compared with that at month 18 (18%) where the median follow up time interval was 7.9 months, suggesting that the shorter interval of 5.1 months was not a contributing factor for those that failed to serorevert at month 6.

At a population level we found there was no difference in the reduction in OD for seropositive participants who had received one dose of ivermectin compared to those who had received two doses. Other studies have reported cure rates of 68% and 70% from serology and 83% and 87% from faecal analysis with a single dose of ivermectin.[5,36–39] The product information from the manufacturer recommends only one dose for uncomplicated *Strongyloides* infection however, one and two dose ivermectin regimens for the treatment of *Strongyloides* have been reported in other studies with cure rates increasing for those administered two doses.[5,39,40]

The importance of *Strongyloides* infections in the Australian Indigenous population is contentious among health professionals and benefits of MDA programs for this infection remain to be elucidated as the degree of morbidity from chronic *Strongyloides* has not been established. The risk of disseminated strongyloidiasis and its association with high mortality[14] however, is not contentious and is managed by hospital and primary health care practitioners implementing the pre-emptive Top End ivermectin therapy guideline for those being prescribed immunosuppressive therapy of a defined nature.[20]

From our questionnaire, symptoms reported on the day that medication was administered showed no correlation with *Strongyloides* results. Notably, non-specific self-reported symptoms of strongyloidiasis (diarrhoea, abdominal pain, skin and respiratory symptoms) on the day medication was administered were not more common in those seropositive. An electronic health record audit of participants from our study is currently being conducted to review the reasons for and frequency of PHC presentations 12 months prior to the study commencing for participants that were seropositive compared with those matched by age and sex that were seronegative at baseline. This may help further elucidate the clinical relevance of *Strongyloides* seropositivity in community members and enable assessment of any relevant findings to compare with the myriad of other community health issues that contribute to the poorer health experienced overall by Aboriginal and Torres Strait Islander people.[41–43].

At baseline and month 12, over 50% of children enrolled aged <5 years were not tested for *Strongyloides* as a faecal specimen was not provided. For children aged 5-<15 years, *Strongyloides* screening increased from 75% at baseline to 89% at month 12 after parents requested we include serological testing of children as well as coprological examination in the protocol. Haemoglobin and eosinophils were not collected at baseline due to budget constraints. However, from month 6 onwards sufficient funds were made available. Not all specimens could be tested for eosinophilia if the sample arrived at WDP after 24–36 hrs of being collected. A field testing station to prepare the blood samples for transportation and perform coprological examinations on faecal specimens was established in the community. This field testing station did not have the resources or funds to be able to perform coprological examination on all enrolled participants.

Eosinophilia was associated with seropositivity however, almost half of those seronegative also had an eosinophilia. Scabies was present in approximately one fifth of participants at month 12 that were seronegative with eosinophilia (baseline cohort 43/198, 21% and new entrants 16/79, 20%) but we were unable to determine if other helminth infections were contributing to the eosinophilia as faecal specimens were only collected for testing children for whom we did not get a venous blood. Worldwide, eosinophilia is most commonly caused by helminth infections,[44] which are still prevalent in the region where this study was conducted.[45,46] However, over 40% of the baseline cohort and 11% of new entrants that were seropositive had no eosinophilia, indicating that the absence of eosinophilia does not exclude the possibility of a helminth infection. The significantly reduced eosinophilia in the baseline cohort at month 12 compared to the new entrants may have been attributable to the MDA at baseline however, as eosinophilia was not tested for at baseline this cannot be confirmed.

In Central Australia, where the link between endemic HTLV-1 infection and clinical disease from strongyloidiasis has been studied,[16] the introduction of an ivermectin MDA is likely to have clinical benefits. Whilst an MDA for *Strongyloides* may not in itself be a priority for the Top End of the Northern Territory, the success of this ivermectin MDA in reducing and sustaining a low *Strongyloides* seroprevalence over 18 months provides evidence that it has the potential to be an effective public health measure. Assessment of the impact of such a program on the regional prevalence of *Strongyloides* and its sustainability could take into consideration the potential for regional eradication and also the impact of ivermectin on other infections such as scabies. The use of dried blood spots to define the antibody response to *S*. *stercoralis* recombinant antigen NIE provides a non-invasive collection method to accurately determine seroprevalence, particularly in children.[47]

Of note, the role of MDAs for endemic *Strongyloides* and of individual therapy for non-immunosuppressed asymptomatic individuals in endemic communities has recently been challenged by a study suggesting *Strongyloides* may have a protective benefit against development of type 2 diabetes (T2DM) and metabolic syndrome in the Australian Indigenous population.[48] This is supported by a limited body of evidence on animal models[49] and humans[50] that suggest helminth infections are able to attenuate the development of metabolic disorders such as T2DM.[51]

The reduction in *Strongyloides* prevalence in this study was reassuring in confirming that a program built on community engagement and education in combination with an ivermectin MDA can have a positive impact on reducing prevalence. The study also supported the need for MDAs to be repeated, in this case yearly, when there is substantial movement of untreated people into the community. The logical extension of the findings from this study is MDAs involving larger populations to encompass whole regions within which population movements occur. In summary, two community ivermectin MDAs delivered 12 months apart by trained Aboriginal researchers in collaboration with non-Indigenous researchers resulted in a sustained and significant reduction in *Strongyloides* seroprevalence over 18 months. Ongoing studies are required to clarify the benefits and any potential harms of MDAs for the differing epidemiological circumstances seen globally for *Strongyloides* and other geohelminth infections.

## Supporting information

S1 Checklist CONSORT 2010 checklist of information to include when reporting a randomised trial. (DOC)

S1 Data Supplementary data for month 6 prevalence calculation. (DOCX)

S2 Data Supplementary data for month 18 prevalence calculation of the baseline cohort. (DOCX)

S3 Data Supplementary data for month 18 prevalence calculation of the new entrants at month 12. (DOCX)

S1 Fig Optical density at baseline and month 6 survey for participants (n = 3) that received 1 dose of ivermectin at baseline and failed to serorevert, (*Strongyloides* positive >0.45). (TIF)

S2 Fig Optical density at baseline and month 6 survey for participants (n = 20) that received 2 doses of ivermectin at baseline and failed to serorevert, (*Strongyloides* positive >0.45). (TIF)

S3 Fig Optical density at month 12 and month 18 survey for participants (n = 3) that received 1 dose of ivermectin at month 12 and failed to serorevert, (*Strongyloides* positive >0.45). (TIF)

S4 Fig Optical density at month 12 and month 18 survey for participants (n = 15) that received 2 doses of ivermectin at month 12 and failed to serorevert, (*Strongyloides* positive >0.45). (TIF)
