# Supplementary material for: Strongyloides seroprevalence before and after an ivermectin mass drug administration in a remote Australian Aboriginal community
Source: PLoS Negl Trop Dis. 2017 May 15;11(5):e0005607. doi: 10.1371/journal.pntd.0005607 (PMC5444847; doi:10.1371/journal.pntd.0005607)
Supplement: S1 Data — (DOCX) [file pntd.0005607.s002.docx]

**S1 - Supplementary data for month 6 prevalence calculation.**

The numbers in red are participants seen at the month 6 survey who had also been seen at the baseline population census six months prior. The numbers in black brackets [..] are the participants seen in each category from the baseline population census. The red denominator in the second column and second row (*Strongyloides* seronegative*,* scabies absent) are participants that were seen (n=141) from a list of 200 randomly selected participants, for whom we were aiming to screen 160 who were negative for both scabies and *Strongyloides*. The figures in black brackets [..] in the third and fourth column (*Strongyloides* equivocal and seropositive) and third row (scabies present) are those that were to be followed up at the month 6 survey. Not all participants that were to be followed up were able to be located for review at month 6.

**Table A. *Strongyloides* serostatus at month 6 / participants seen at month 6 [participants seen at month 0], by scabies status and *Strongyloides* serostatus at month 0.**

|  | *Strongyloides* seronegative baseline | *Strongyloides* equivocal  baseline | *Strongyloides* seropositive baseline | *Strongyloides* unknown baseline | Total |
| --- | --- | --- | --- | --- | --- |
| Scabies absent baseline | 4/141 (3%)  [500] | 6/83 (7.2%)  [114] | 23/127  [167] | 0  [179] | 33/351 (9.4%)  [960] |
| Scabies present  baseline | 0/16  [19] | 0/4  [6] | 0/7  [ 8] | 2/8  [9] | 2/35 (5.7%)  [42] |
| Scabies unknown  baseline | 0/0  [3] | 0/1  [1] | 0/0  [7] | 0/0  [0] | 0/1  11 |
| Total | 4/157 (2.5%)  [522] | 6/88 (6.8%)  [121] | 23/134 (17%)  [175] | 2/8 (25%)  [195]* | 35/387 (9.0%)  [1,013] |

*Note. Eight extra household contacts were examined at month 6 (not included in the above) of which five had serology performed, one was found to be positive, one equivocal and three negative for Strongyloides.*

**Includes 41 participants that provided a faecal specimen of which four were positive but their Strongyloides serostatus was unknown*.

- Prevalence baseline: 175/818 = 21% (195 participants had missing *Strongyloides* serology)
- Failure to serorevert at month 6: 23/134 (17%) with positive *Strongyloides* serology at baseline failed to serorevert at month 6
- Positive *Strongyloides* seroconversions at month 6: 4/157 (2.5%) with negative *Strongyloides* at baseline had a positive seroconversion at month 6
- Prevalence month 6: (23/134)*175 + (4/157)*522]/818= 43/818 = 5%
