# Supplementary material for: Strongyloides seroprevalence before and after an ivermectin mass drug administration in a remote Australian Aboriginal community
Source: PLoS Negl Trop Dis. 2017 May 15;11(5):e0005607. doi: 10.1371/journal.pntd.0005607 (PMC5444847; doi:10.1371/journal.pntd.0005607)
Supplement: S2 Data — (DOCX) [file pntd.0005607.s003.docx]

**S2 - Supplementary data for month 18 prevalence calculation of the baseline cohort.**

The numbers in red are baseline participants seen at the month 18 survey who had also been seen at the month 12 population census. The numbers in black brackets [..] are the baseline participants seen in each category at the month 12 population census. The red denominator in the second column and second row (*Strongyloides* seronegative*,* scabies absent) are baseline participants that were seen (n=89) from a list of 200 randomly selected participants from the baseline and new entrant cohorts, from which we were aiming to screen 160 who were negative for both scabies and *Strongyloides*. The figures in black brackets [..] in the third and fourth column (*Strongyloides* equivocal and seropositive) and third row (scabies present) are those that were to be followed up at the month 18 survey. Not all baseline participants that were to be followed up were able to be located for review at the month 18 survey.

**Table B. Baseline Cohort - *Strongyloides* serostatus at month 18 / participants seen at month 18 [participants seen at month 12], by scabies status and *Strongyloides* serostatus at month 12.**

|  | *Strongyloides* seronegative month 12 | *Strongyloides*  equivocal month 12 | *Strongyloides* seropositive month 12 | *Strongyloides* unknown month 12 | Total |
| --- | --- | --- | --- | --- | --- |
| Scabies absent month 12 | 1/89 (11%)  [478] | 7/42 (17%)  [56] | 9/26 (32%)  [32] | 0/1  [71] | 17/158 (11%)  [637] |
| Scabies present  month 12 | 0/31  [43] | 0/4  [7] | 0/2  [2] | 0/1  [11] | 0/38  [63] |
| Scabies unknown  month 12 | 0/0  [0] | 0/0  [0] | 0/0  [0] | 0/0  [0] | 0/0  [0] |
| Total | 1/120 (0.8%)  [521] | 7/46 (15%)  [63] | 9/28 (32%)  [34] | 0/2  [82]* | 17/196 (9%)  [700] |

*Note. Fifteen household contacts were examined at month 18 (not included in the table above) of which one was equivocal for strongyloidiasis, one faecal microscopy/culture negative, seven seronegative and six serostatus was unknown.*

**Includes 18 participants that provided a faecal specimen at month 12 of which one was positive but their Strongyloides serostatus was unknown*.

- There were 238 baseline cohort participants seen at month 18, excluded from the analysis were 27 participants that were not tested for *Strongyloides* and 15 household contacts.
- Prevalence month 12: 34/618 = 6% (82 participants had missing *Strongyloides* serology)
- Failure to serorevert at month18: 9/28 (32%) with positive *Strongyloides* serology at month 12 failed to serorevert at month 18
- Positive *Strongyloides* seroconversions at month 18: 1/120 (1%) with negative *Strongyloides* at month 12 had a positive seroconversion at month 18
- Prevalence month 18: [(9/28)*34 + (1/120)*521]/618 = 15/618 = 2%
