# Supplementary material for: Strongyloides seroprevalence before and after an ivermectin mass drug administration in a remote Australian Aboriginal community
Source: PLoS Negl Trop Dis. 2017 May 15;11(5):e0005607. doi: 10.1371/journal.pntd.0005607 (PMC5444847; doi:10.1371/journal.pntd.0005607)
Supplement: S3 Data — (DOCX) [file pntd.0005607.s004.docx]

**S3 - Supplementary data for month 18 prevalence calculation of the new entrants at month 12.**

The numbers in red are new entrants seen at the month 18 survey who had also been seen at the month 12 population census. The numbers in black brackets [..] are the new entrants seen in each category from the month 12 population census. The red denominator in the second column and second row (*Strongyloides* seronegative*,* scabies absent) are participants that were seen (n=33) from a list of 200 randomly selected participants that were negative for both *Strongyloides* and scabies from the baseline and new entrant cohorts, from which we were aiming to screen 160 participants. The figures in black brackets [..] in the third and fourth column (*Strongyloides* equivocal and positive) and third row (scabies present) are the new entrants that were to be followed up at the month 18. Not all new entrants that were to be followed up were able to be located for review at the month 18 survey.

**Table C. New entrants at month 12 - *Strongyloides* serostatus at month 18 / new entrants seen at month 18 [new entrants seen at month 12], by scabies status and *Strongyloides* serostatus at month 12.**

|  | *Strongyloides* seronegative month 12 | *Strongyloides*  equivocal month 12 | *Strongyloides* seropositive month 12 | *Strongyloides* unknown month 12 | Total |
| --- | --- | --- | --- | --- | --- |
| Scabies absent month 12 | 0/33  [165] | 3/26 (12%)  [38] | 9/41(22%)  [62] | 0/0  [45] | 12/100 (12%)  [310] |
| Scabies present  month 12 | 2/10 (20%)  [16] | 0/3  [3] | 0/9  [13] | 0/3  [18] | 2/25 (8%)  [50] |
| Scabies unknown  month 12 | 0/0  [0] | 0/0  [0] | 0/0  [0] | 0/0  [0] | 0/0  [0] |
| Total | 2/43 (5%)  [181] | 3/29 (10%)  [41] | 9/50 (18%)  [75] | 0/3  [63] | 14/125 (11%)  [360] |

*Note. Five household contacts were examined at month 18 (not included in the table above) one was negative and the other four were not tested for Strongyloides.*

- There were 150 new entrants seen at month 18, excluded from the analysis were 20 participants that were not tested for *Strongyloides* and five household contacts.
- Prevalence month 12: 75/297 = 25% (63 participants had missing *Strongyloides* serology)
- Failure to serorevert at month18: 9/50 (18%) with *Strongyloides* at month 12 still had *Strongyloides* at month 18
- Positive *Strongyloides* seroconversions at month 18: 2/43 (5%) with no *Strongyloides* at month 12 had a positive seroconversion at month 18
- Prevalence month 18: [(9/50)*75 + (2/43)*181]/297 = 22/297 = 7%
