# Supplementary figures and images for: Strongyloides seroprevalence before and after an ivermectin mass drug administration in a remote Australian Aboriginal community
Source: PLoS Negl Trop Dis. 2017 May 15;11(5):e0005607. doi: 10.1371/journal.pntd.0005607 (PMC5444847; doi:10.1371/journal.pntd.0005607)

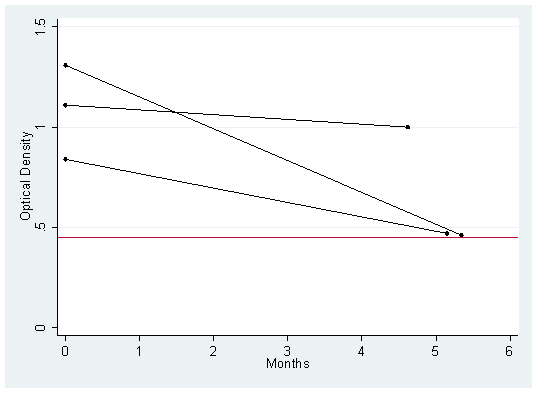

Supplement: S1 Fig — (TIF) [file pntd.0005607.s005.tif]

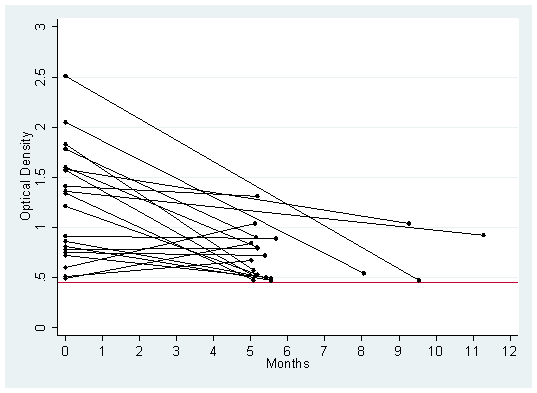

Supplement: S2 Fig — (TIF) [file pntd.0005607.s006.tif]

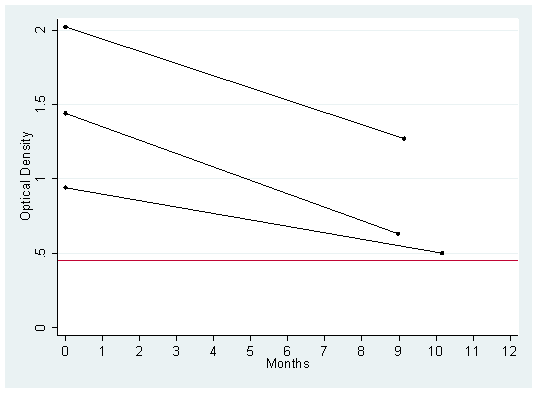

Supplement: S3 Fig — (TIF) [file pntd.0005607.s007.tif]

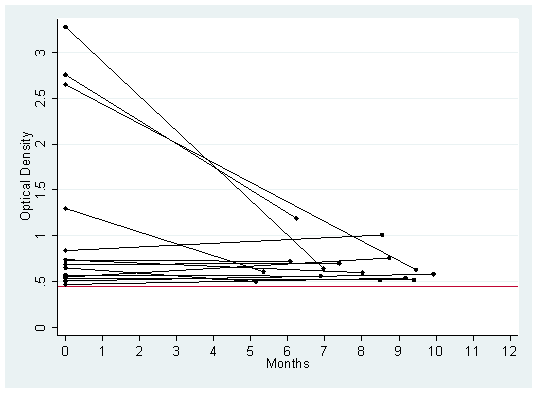

Supplement: S4 Fig — (TIF) [file pntd.0005607.s008.tif]
